# Data Mining and Expression Analysis of Differential lncRNA ADAMTS9-AS1 in Prostate Cancer

**DOI:** 10.3389/fgene.2019.01377

**Published:** 2020-02-21

**Authors:** Jiahui Wan, Shijun Jiang, Ying Jiang, Wei Ma, Xiuli Wang, Zikang He, Xiaojin Wang, Rongjun Cui

**Affiliations:** ^1^ Department of Biochemistry and Molecular Biology, Mudanjiang Medical University, Mudanjiang, China; ^2^ Department of Clinical Laboratory, Harbin Public Security Hospital, Harbin, China; ^3^ Department of Clinical Laboratory, Daqing Medical College, Daqing, China; ^4^ Department of Clinical Laboratory, The Seventh Hospital in Qiqihar, Qiqihar, China

**Keywords:** prostate cancer, ADAMTS9-AS1, ceRNA, Hsa-miR-96, PRDM16

## Abstract

Long noncoding RNAs (lncRNAs) play important roles in the regulation of gene expression by acting as competing endogenous RNAs (ceRNAs). However, the roles of lncRNA-associated ceRNAs in oncogenesis are not fully understood. The present study aims to determine whether a ceRNA network can serve as a prognostic marker in human prostate cancer (PCa). In order to identify a ceRNA network and the key lncRNAs in PCa, we constructed a differentially expressed lncRNAs (DELs)–differentially expressed miRNAs (DEMis)–differentially expressed mRNAs (DEMs) regulatory network based on the ceRNA theory using data from the Cancer Genome Atlas (TCGA). We found that the DELs–DEMis–DEMs network was composed of 27 DELs nodes, seven DEMis nodes, and three DEMs nodes. The 27 DELs were further analyzed with several public databases to provide meaningful information for understanding the functional roles of lncRNAs in regulatory networks in PCa. We selected ADAMTS9-AS1 to determine its role in PCa and found that ADAMTS9-AS1 significantly influences tumor cell growth and proliferation, suggesting that it plays a tumor suppressive role. In addition, ADAMTS9-AS1 functioned as ceRNA, effectively becoming a sponge for hsa-mir-96 and modulating the expression of PRDM16. These results suggest that ceRNAs could accelerate biomarker discovery and therapeutic strategies for PCa.

## Introduction

Prostate cancer (PCa) is a common malignancy of the urinary and reproductive systems. Since the late 1980s and early 1990s, the incidence and mortality of PCa have soared worldwide (Bray et al., 2013; Smith et al., 2017). The Cancer Statistics report has revealed an expected 174,650 new cases of PCa and 31,620 deaths in the United States (Siegel et al., 2019). In China, poor dietary habits including excessive consumption of animal fat and reduced fiber intake have led to an increasing number of PCa patients. PCa ranks 7th in incidence and 10th in mortality among malignant tumors in China (Zhang et al., 2017). Due to the lack of obvious symptoms during early-stage PCa, it is often overlooked, which results in reduced treatment success. Unfortunately, the molecular mechanisms underlying PCa metastasis are not known and thus relevant targets to effectively predict PCa progression are lacking. Therefore, investigating the molecular mechanisms of PCa has gained much attention.

Long noncoding RNAs (lncRNAs) are longer than 200 nucleotides in length and are of much recent interest (Ling et al., 2015). LncRNAs play important roles in cancer, such as chromatin remodeling and transcriptional and post-transcriptional regulation (Fatima et al., 2015; Yarmishyn and Kurochkin, 2015; Wu and Hann, 2018). The most recognized molecular mechanism of lncRNA is to act as a microRNA “sponge” that regulates the activity of mRNAs, and thus lncRNAs are also referred to as competing endogenous RNAs (ceRNAs) (Salmena et al., 2011; Karreth et al., 2015). ceRNAs are involved in the pathology of various tumors, including colorectal cancer (Chen et al., 2017), breast cancer (Wang et al., 2017), and non-small cell lung cancer (Qu et al., 2017). However, the role of ceRNAs in PCa remains unclear.

## Materials and Methods

### Raw Data

To investigate the significance of lncRNAs, miRNAs, and mRNAs in PCa, we downloaded gene expression data from the TCGA project webpage (https://www.cancer.gov/about-nci/organization/ccg/research/structural-genomics/tcga), including 499 PCa samples and 52 matched normal samples up to April 9th, 2018.

### Screening Differentially Expressed lncRNAs, miRNAs, and mRNAs

TCGA RNA-Seq raw data was performed with package edgeR. The P-value was set at 0.01 and the log2 fold change was set at 2. The data was presented as a heatmap plot and volcano map. Based on the analysis of DELs, DEMis, and DEMs, a correlation analysis was conducted on each significant DELs, DEMis, and DEMs.

### DELs–DEMis–DEMs Network

Based on the relationship between DELs, DEMis, and DEMs, the ceRNA network was constructed in three steps: 1) PCa-specific RNA (DELs, DEMis, and DEMs) filtration: to maximize data reliability, gene matrix was converted to gene ID by Ensembl (http://asia.ensembl.org/index.html); 2)DELs-DEMis interactions were predicted using miRcode (http://www.mircode.org/); 3) miRDB (http://www.mirdb.org/), miRTarBase (http://mirtarbase.mbc.nctu.edu.tw/), and Targetscan (http://www.targetscan.org/) were used to predict the DEMs targeted by DEMis. Furthermore, DEMis that regulated the expression of both DELs and DEMs were selected for construction of the ceRNA network. The ceRNA relationships were integrated using an in-house Perl script and R package. Cytoscape v3.0 was used to construct and visualize the ceRNA network.

### Database and Functional Enrichment Analysis

The online database GEPIA (http://gepia.cancer-pku.cn) is an interactive web server for analyzing RNA sequencing expression based on TCGA and GTEx projects. We obtained the significant lncRNA ADAMTS9-AS1 in PCa *via* the database. The gene ontology (GO) analysis was performed for the functional annotation of ADAMTS9-AS1. The pathways that ADAMTS9-AS1 mainly participated in were investigated by KEGG pathway analysis. GO terms and pathways with a P value < 0.01 were considered significant. Both GO and KEGG pathway analyses were carried out in the Database for circlncRNAnet (http://app.cgu.edu.tw/circlnc/).

### Cell Culture

The human PCa cell line DU145 was obtained from the Cell Bank of the Chinese Academy of Science (Shanghai, China). The cell lines were cultured in RPMI1640 medium supplemented with 10% fetal bovine serum (Gibco), 100 U/ml penicillin, and 100 mg/ml streptomycin (Gibco) at 37°C in humidified air containing 5% of CO_2_.

### RNA Extraction and Quantitative PCR

Total RNA was isolated using Trizol reagent (Invitrogen). First strand cDNA was generated using the Reverse EasyScript One Step gDNA Removal and cDNA Synthesis SuperMix (Trangene). qRT-PCR was performed using SYBR Green Master Mixture (Roche) reagent in an ABI7500 real-time PCR instrument. GAPDH was used as an internal control. The relative levels of gene expression were calculated by the 2^−ΔΔCt^ method. qRT-PCR primers were as follows: ADAMTS9-AS1 forward 5’-CTCAGACCACAACTCTCCACCTTG-3’, reverse 5’-CAGATGCTGCCTGGCTGATGG-3’; PRDM16 forward: 5’-ATGTATGAGCCCAACCGGGA-3’, reverse 5’-AGCTCGAAGTCTGCTGGGAT’; GAPDH forward 5’-GAAGGTCGGAGTCAACGGATT-3’, reverse 5’-CGCTCCTGGAAGATGGTGAT-3’. All experiments were performed in triplicate.

### Cell Transfection

Small interfering siRNAs specifically targeting ADAMTS9-AS1 were synthesized by Shanghai Gene Pharma Co, Ltd. siRNA sequences for ADAMTS9-AS1: siRNA 1, 5′-GGAATTCAAGCTTCTACAA-3′; siRNA 2, 5′-CCACTGAACACATAAACAT-3′; siRNA 3, 5′-GGACTTGCAACTGTGACTT-3′; negative control: 5′-UUCUCCGAACGUGUCACGUTT-3′. siRNA plasmids were transfected into cells using Lipofectamine TM 2000 (Invitrogen, Carlsbad, CA, USA) and were incubated for 24 h according to the manufacturer’s instructions.

### Cell Proliferation Assay

Cell proliferation was assessed using the Cell Counting Kit-8 (Dojindo) according to the manufacturer’s protocol. Brieﬂy, 1 × 10^5^ cells were seeded into each well of 24-well plates. Before proliferation assessment, CCK-8 reagent (30 μl) and phenol-free RPMI-1640 medium (300 μl) were added to each well, and incubated at 37°C for 24, 48, 72, and 96 h. Viable cells were evaluated by absorbance measurements at 450 nm at each time point. Each experiment was performed in triplicate and repeated three times.

### Dual Luciferase Reporter Assay

To verify the binding site of ADAMTS9-AS1-hsa-mir-96-RDM16, the fragment of ADAMTS9-AS1 containing the predicted hsa-mir-96 binding site and the 3’UTR of RDM16 were amplified from human cell genomic DNA and then cloned into psi-CHECK-2 vector (Promega, Madison, WI). Mutant plasmids were generated by deleting the predicted binding site. The appropriate plasmid and hsa-mir-96 mimic or negative control were co-transfected into HEK293T cells (1.0 × 10^5^), and the luciferase assay was evaluated 48 h after transfection using the dual-luciferase reporter assay system (Promega). Renilla luciferase activity was used as a control.

### Statistical Analysis

Data are presented as mean ± standard deviation (SD). T-tests were used to measure statistically significant differences. The Pearson’s correlation coefficient was used to analyze the correlation between ADAMTS9-AS1 in cancer tissues. Survival plots were generated by Kaplan–Meier analysis, and the log-rank test was used to assess statistical significance. *P* < 0.05 was considered statistically significant. All statistical analyses were performed using GraphPad Prism 5.0 (GraphPad Software, Inc., La Jolla, CA). Each experiment was performed three times.

## Results

### Identification of Significant DELs, DEMis, and DEMs

**Figure 1:**
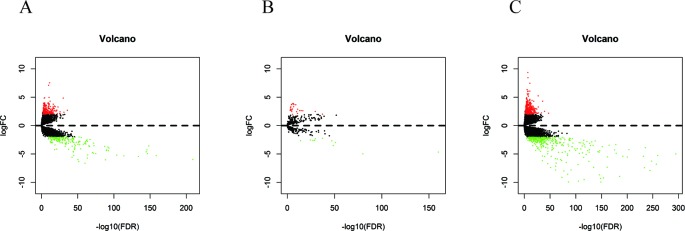
Volcano plots of DELs **(A)**, DEMis **(B)**, and DEMs **(C)** in PCa and adjacent normal prostate tissue.

In 499 PCa patients from the TCGA database, we initially performed differential expression analysis by comparing the expression of 14,447 lncRNAs in PCa and adjacent normal prostate tissue. The edgeR package (fold change >2, *P* < 0.01) identified 381 DELs (215 upregulated and 166 downregulated) (**Supplementary Table 1**) in PCa and adjacent normal prostate tissue. A total of 500 miRNAs and 19,676 mRNAs were identified and 35 miRNAs (24 upregulated and 11 downregulated) and 689 mRNAs (333 upregulated and 556 downregulated) were found to be differentially expressed between PCa and adjacent normal prostate tissue (fold change >2, *P* < 0.01) (**Supplementary Tables 2** and **3**). The distribution of all the significant DELs, DEMis, and DEMs is shown in a volcano map in **Figure 1** and a heat map is shown in **Supplementary Figure S1**.

### DEMis Targeted by DELs

As we found 381 DELs, miRcode and Perl were used to predict DELs-targeted DEMis in the 381 DELs and indicated that 27 DELs might target seven DEMis (**Table 1**).

**Table 1:** DEMis targeted by DELs.

DELs	DEMis
KIAA0087	hsa-mir-96
C5orf64	hsa-mir-184 hsa-mir-122 hsa-mir-506
LINC00308	hsa-mir-137
LINC00313	hsa-mir-372 hsa-mir-187 hsa-mir-122
LINC00336	hsa-mir-96 hsa-mir-506
AC092811.1	hsa-mir-96
UCA1	hsa-mir-96 hsa-mir-184 hsa-mir-122 hsa-mir-506
PCA3	hsa-mir-96 hsa-mir-137
LINC00355	hsa-mir-122 hsa-mir-506
HCG22	hsa-mir-96 hsa-mir-122 hsa-mir-506
XIST	hsa-mir-372 hsa-mir-96 hsa-mir-137 hsa-mir-122
EMX2OS	hsa-mir-184 hsa-mir-506
AL161645.1	hsa-mir-96 hsa-mir-184 hsa-mir-122
NALCN-AS1	hsa-mir-372 hsa-mir-506
ERVH48-1	hsa-mir-96 hsa-mir-137 hsa-mir-184 hsa-mir-187
OSTN-AS1	hsa-mir-137 hsa-mir-506
DSCAM-AS1	hsa-mir-137 hsa-mir-122
GPC5-AS1	hsa-mir-372
ZBTB20-AS3	hsa-mir-122 hsa-mir-506
AL356133.2	hsa-mir-372
ADAMTS9-AS1	hsa-mir-96
HNF1A-AS1	hsa-mir-372 hsa-mir-122
AL353803.1	hsa-mir-122
ALDH1L1-AS2	hsa-mir-372
LNX1-AS2	hsa-mir-506
PCAT1	hsa-mir-372 hsa-mir-122 hsa-mir-506
ANO1-AS2	hsa-mir-372

### DEMs Targeted by DEMis

**Figure 2:**
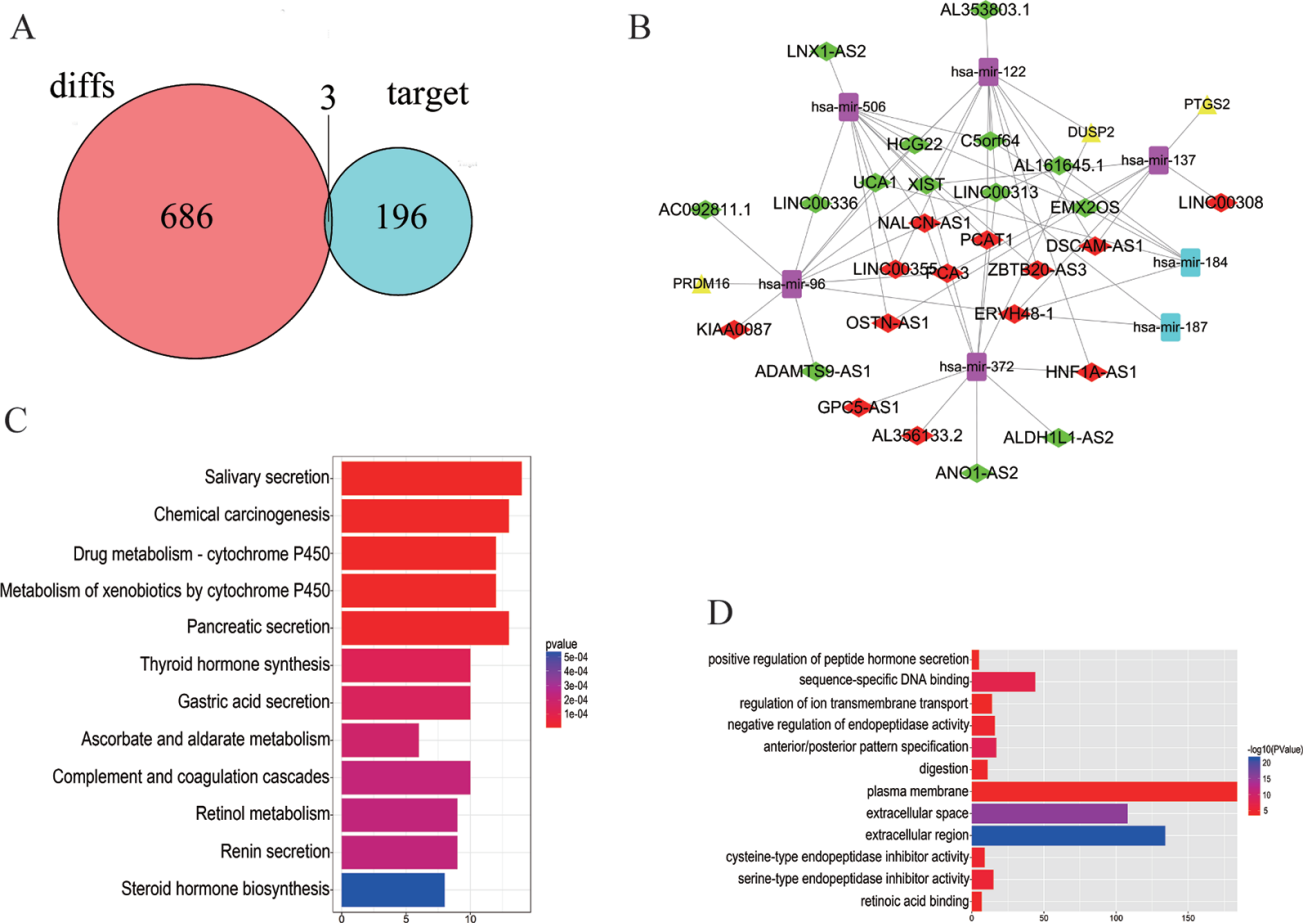
Distribution of differentially expressed genes. **(A)** 686 DEMs, 196 DEMis-targeted DEMs, and three DELs that intersect with DEMs and DEMis-targeted DEMs were found. **(B)** The DELs–DEMis–DEMs regulator network in PCa contains 27 DELs nodes, seven DEMis nodes, and three DEMs nodes. Down DELs (*green rhombus*), up DELs (*red rhombus*), up DEMs (*yellow triangle*), up DEMis (*purple square*), down DEMis (*blue square*). **(C**, **D)** The KEGG pathway and GO enrichment analyses with the 12 most significant p-values. The x-axis denotes the number of DEmRNAs involved in the pathway.

The 7 DEMis discovered included hsa-mir-96, hsa-mir-184, hsa-mir-122, hsa-mir-506, hsa-mir-137, hsa-mir-372, and hsa-mir-187. Perl was used to modify DEMis 3p and 5p by starBase (**Table 2**). Subsequently, miRDB, miRTarBases, and TargetScan were used to predict DEMis-targeted DEMs (**Table 3**). Then, the edgeR package identified three DEMis-targeted DEMs and differentially expressed mRNA, including PRDM16, PTGS2, and DUSP2 (**Figure 2A**).

**Table 2:** The modification of DEMis 3p and 5p.

miRNA	miRNAp
hsa-mir-137	hsa-mir-137
hsa-mir-184	hsa-mir-184
hsa-mir-187	hsa-mir-187-3p
hsa-mir-122	hsa-mir-122-5p
hsa-mir-372	hsa-mir-372-3p
hsa-mir-96	hsa-mir-96-5p
hsa-mir-506	hsa-mir-506-3p

**Table 3:** DEMs targeted by DEMis.

miRNA	Gene
hsa-mir-122-5p	RBM43 HECTD3 P4HA PKM SLC7A1 ALDOA G6PC3 PRKRA RBL1 CLIC4 NT5C3A NPEPPS AKT3 GALNT3 ANKRD13C CCNG1 TGFBRAP1 GYS1 FAM117B ORC2 PIP4K2A SLC9A1 SLC52A2 BROX NFX1 DUSP2 PHF14 TNRC6A FUNDC2 HECW2 GNPDA2 CCDC43
hsa-mir-137	GLO1 RREB1 CTBP1 ZNF326 SFT2D3 AGO4 KDM1A LIMCH1 MITF YBX1 E2F6 KIT PTGS2 PAPD7 EOGT DR1 YTHDF3 RORA ESRRA FMNL2 NCOA3 CSE1L SLC1A5 GIGYF1 NCOA2 PXN GLIPR1 HNRNPDL SNRK
hsa-mir-184	LRRC8A
hsa-mir-187-3p	DYRK2
hsa-mir-372-3p	SERF1A ZFYVE26 TNKS2 MED17 WEE1 ULK1 TAOK1 LIMA1 CLIP4 REST NR2C2 BTG1 ZNF532 GALNT3 HABP4 TMEM100 TNFAIP1 MINK1 FAM102B MIXL1 CPT1A DUSP2 TMEM19 MPP5 SUCO PSD3 KPNA2 CREBRF RAB11FIP1 DPP8 RHOC PAK2 SAR1B NFIB TRPS1 SBNO1 LEFTY1 SH3GLB1 SLC7A11 ZNF385A PLA2G12A SIK1 UNK FBXL7 CADM2 IGF1R MBNL2 SUZ12 MKNK2 GNB5 TFAP4 FEM1C FOXJ2 ELAVL2 TIMM17A PFKP TGFBR2 HIP1 KREMEN1 ATAD2 CCSAP RAB22A KLF3 LATS2 CUL3 INO80D IRF2 HMBOX1 SLC22A23 ELK4 OSTM1 YOD1 SLAIN2 SERF1B IRAK4 TXNIP PTPDC1 ARID4B DAZAP2
hsa-mir-506-3p	TMEM41A CREBRF SCAMP4 CD151 LRRC58 CHSY1 PTBP3 NEK9 NUFIP2 AMOTL1 LRRC1 SFT2D3 PTBP1 SNAI2 VIM MYO10 SNX18 PI4K2B GXYLT1 PARP16 PRR14L ZWINT SLC16A1
hsa-mir-96-5p	CNNM3 EDEM1 SCARB1 PRDM16 ZEB1 PROK2 APPL1 NHLRC3 SLC39A1 TSKU MED1 CASP2 MBD4 CCNG1 ADCY6 TMEM170B PPP1R9B DDIT3 PRKCE MAP3K3 EIF4 EBP2 REV1 DDAH1 ALK ABCD1 SIN3B SLC1A1 TRIB3 PRKAR1A SNX16 SLC25A25 STK17B MORF4L1 FRS2 SNX7 ASH1L JAZF1 KRAS FOXO1

### DELs–DEMis–DEMs Network

To better understand the functions of lncRNAs acting as miRNA targets, a network among DELs, DEMis, and DEMs was first constructed and then visualized. The DELs–DEMis–DEMs network was composed of 27 DELs nodes, seven DEMis nodes, and three DEMs nodes (**Figure 2B**). To explore the pathway analysis of lncRNA, the mRNAs of the ceRNA network in PCa were analyzed by KEGG pathways and GO terms. Next, the top 12 most significant KEGG pathways and the 12 most significantly enriched GO terms were selected (**Figures 2C, D**). Several of these pathways are reported to be involved in the pathogenesis of cancers, including positive regulation of peptide hormone (Liu et al., 2018), sequence-specific DNA binding (Nardini et al., 2013), salivary secretion (Pedersen et al., 2018), and chemical carcinogenesis (Poirier, 2016).

### ADAMTS9-AS1 Is a Potential Prognostic Biomarker of PCa

**Figure 3:**
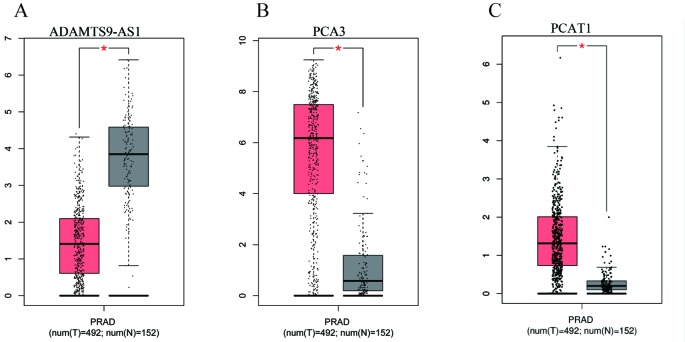
Box plots of three DELs. The cases were divided into a high and low expression group by mean DEL level (*P < 0.05). **(A)** ADAMTS9-AS1, **(B)** PCA3, **(C)** PCAT1.

**Figure 4:**
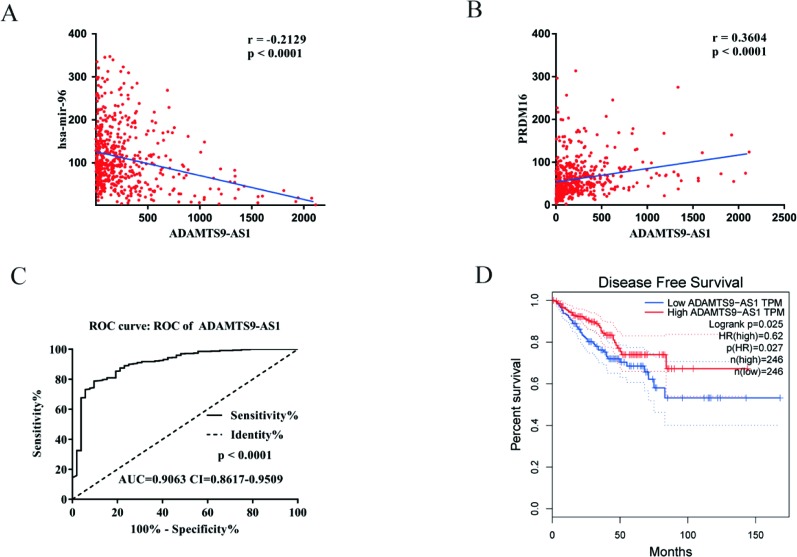
Clinical significance of ADAMTS9-AS1. **(A)** The expression of ADAMTS9-AS1 was negatively correlated with hsa-mir-96 in PCa based on the TCGA dataset. **(B)** The expression of ADAMTS9-AS1 was positively correlated with PRDM16 in PCa based on the TCGA dataset. **(C)** ROC curves of ADAMTS9-AS1 sorted by AUC in PCa; sensitive curve (*solid line*) and identity curve (*dotted line*). The X axis denotes the false positive rate: “1-Specificity”. The Y axis denotes the true positive rate: “Sensitivity”. These curves were provided by GraphPad Prism6. **(D)** Disease-free survival analysis of ADAMTS9-AS1 with low and high ADAMTS9-AS1 TPM. The X axis denotes disease-free survival time (months) and the Y axis denotes the survival rate. These curves were generated by GEPIA.

To find a potential prognostic lncRNA, the 27 DELs obtained from TCGA were used for further analysis with the database GEPIA. Only three DELs exhibited significant prognostic value for PCa, including ADAMTS9-AS1, PCA3, and PCAT1 (**Figure 3**). Moreover, recent accumulating evidence has demonstrated that PCAT1 and PCA3 play important roles in the regulation of gene expression by acting as ceRNAs in PCa (Prensner et al., 2014; Jeske et al., 2017). Significant ceRNA correlations were observed between ADAMTS9-AS1, PRDM16, and hsa-mir-96. Original data on ADAMTS9-AS1, PRDM16, and hsa-mir-96 was extracted from the TCGA platform (**Figures 4A, B**). ADAMTS9-AS1 exhibited high diagnostic value for distinguishing PCa from non-cancer prostate tissues with an AUC of 0.9063 (**Figure 4C**). More interestingly, disease-free survival analysis of ADAMTS9-AS1 with a low TPM exhibited poorer survival compared to ADAMTS9-AS1 with a high TPM (P = 0.0025, **Figure 4D**). These results suggest that ADAMTS9-AS1 may be a potential biomarker for PCa.

### Functional Assessment of ADAMTS9-AS1

GO enrichment and KEGG pathway analyses of ADAMTS9-AS1 were conducted using circlncRNAnet. GO enrichment may directly reflect the distribution of ADAMTS9-AS1 for each enriched GO term of a significant biological process (BP), cellular component (CC), molecular function (MF), or transcription factor (TF) (**Table 4**). The most significant results of the KEGG pathway enrichment analysis show that ADAMTS9-AS1 is mainly involved in the cGMP-PKG signaling pathway, calcium signaling pathway, and Ras signaling pathway (**Table 5**). GO enrichment and KEGG pathway analyses were closely related to the mechanism of cancer.

**Table 4:** GO terms analyses with the correlative genes of ADAMTS9-AS1.

Category	Term	No. of genes	*p*-value
go_bp	regulation of system process	86	1.01E—13
	multicellular organismal signaling	40	9.32E—10
	developmental growth	79	1.93E—08
	chromosome segregation	50	3.90E—07
	cell-cell signaling	161	8.10E—07
	calcium-mediated signaling	29	1.05E—06
	second-messenger-mediated signaling	39	1.94E—06
	regulation of calcium-mediated signaling	19	1.98E—06
go_cc	cell-substrate junction	76	7.90E—15
	cell-substrate adherens junction	73	1.33E—13
	focal adhesion	72	2.69E—13
	plasma membrane region	125	6.04E—12
	cytoskeleton	225	3.72E—11
	actin cytoskeleton	74	4.08E—11
	integral component of plasma membrane	182	1.26E—09
	proteinaceous extracellular matrix	60	1.57E—09
	intrinsic component of plasma membrane	186	3.57E—09
go_mf	cytoskeletal protein binding	116	2.08E—11
	calcium ion binding	89	1.89E—07
	substrate-specific channel activity	63	2.66E—07
	passive transmembrane transporter activity	66	3.70E—07
	ion channel activity	61	3.74E—07
	channel activity	65	7.28E—07
	actin binding	55	3.0E—06
	glycosaminoglycan binding	35	3.10E—06
go_tf	V$SRF_Q6	73	5.98E—24
	V$SRF_Q5_01	66	1.66E—21
	V$SRF_C	64	3.55E—21
	V$SRF_Q4	65	1.10E—20
	CAGCTG_V$AP4_Q5	210	1.33E—15
	AACTTT_UNKNOWN	236	1.40E—12
	CCAWWNAAGG_V$SRF_Q4	30	2.66E—12
	TGGAAA_V$NFAT_Q4_01	230	5.22E—11
hm	MYOGENESIS	62	5.61E—18
	EPITHELIAL MESENCHYMAL TRANSITION	43	2.84E—07
	UV RESPONSE DN	32	4.90E—06
	G2M CHECKPOINT	39	1.02E—05
	APICAL JUNCTION	34	6.19E—04
	E2F TARGETS	34	7.46E—04

**Table 5:** KEGG pathways analyses with the correlative genes of ADAMTS9-AS1.

KEGG pathways	No. of genes	*p*-value
cGMP-PKG signaling pathway	33	4.58E—08
Calcium signaling pathway	34	1.98E—07
Ras signaling pathway	37	5.91E—06
Adrenergic signaling in cardiomyocytes	25	3.35E—05
cAMP signaling pathway	31	3.44E—05
Rap1 signaling pathway	32	4.49E—05
Proteoglycans in cancer	59	5.64E—05
Pathways in cancer	59	5.67E—04
Wnt signaling pathway	21	1.42E—03
Apelin signaling pathway	20	2.15E—03
MAPK signaling pathway	35	2.48E—03
Hippo signaling pathway - multiple species	7	3.28E—03
Oxytocin signaling pathway	21	3.33E—03
PI3K-Akt signaling pathway	39	4.50E—03
Hedgehog signaling pathway	9	5.34E—03

### ADAMTS9-AS1 Functions as a Sponge for hsa-mir-96 in PC Cells

**Figure 5:**
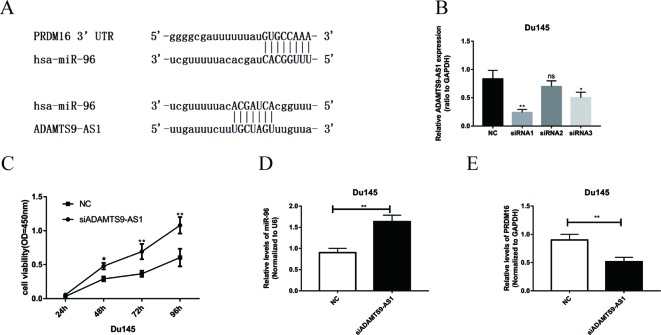
The functions of ADAMTS9-AS1 as ceRNA in PCa cells. **(A)** Complementary binding site of ADAMTS9-AS1, hsa-mir-96, and PRDM16. **(B)** qRT-PCR results of ADAMTS9-AS1 expression levels in DU145 cells transfected with ADAMTS9-AS1-specific siRNA (**P < 0.01). **(C)** CCK-8 assays show that ADAMTS9-AS1 knockdown promoted the proliferation of DU145 cells (**P < 0.01). **(D)** The expression of PRDM16 in DU145 cells following the knockdown of ADAMTS9-AS1 (**P < 0.01). **(E)** hsa-mir-96 expression varied with ADAMTS9-AS1 expression (ns: no significance, *P < 0.05, **P < 0.01).

Bioinformatics methods were used to predict the binding sequence between ADAMTS9-AS1, hsa-mir-96, and PRDM16 (**Figure 5A**). To investigate the biological functions of ADAMTS9-AS1 in PCa cells, we knocked down ADAMTS9-AS1 in DU145 cells by transfecting with specific siRNA. ADAMTS9-AS1 expression was significantly downregulated (P < 0.001) in the si-ADAMTS9-AS1 transfected cells compared to control cells. siRNA1 exhibited the largest downregulation and was thus selected for subsequent experiments (**Figure 5B**). Growth curves from CCK8 proliferation assays showed that ADAMTS9-AS1 knockdown significantly promoted DU145 cell proliferation (**Figure 5C**). These findings suggest that ADAMTS9-AS1 behaves as a tumor suppressor gene that influences PCa cell proliferation. To investigate the molecular mechanism of the ceRNA network between ADAMTS9-AS1, hsa-mir-96, and PRDM16 in PCa, we detected the mRNA level of hsa-mir-96 and PRDM16 in si-ADAMTS9-AS1 PCa cells. We found that knockdown of ADAMTS9-AS1 also significantly reduced PRDM16 mRNA levels; however, hsa-mir-96 expression was increased in DU145 cells (**Figures 5D, E**). These results suggest that ADAMTS9-AS1 functions as a ceRNA for hsa-mir-96, thereby leading to the regulation of its endogenous target PRDM16.

### ADAMTS9-AS1 Regulated PRDM16 Expression Indirectly by Sponging hsa-mir-96

**Figure 6:**
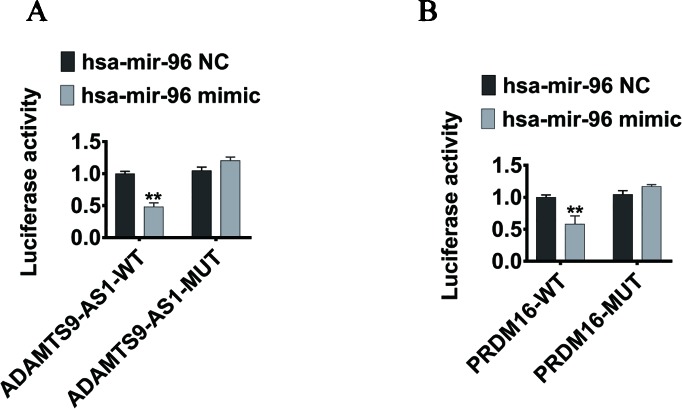
PRDM16 is an hsa-mir-96 target gene and is indirectly regulated by ADAMTS9-AS1. **(A)** The luciferase reporter plasmid containing wild type (AC-WT) or mutant (AC-MUT) ADAMTS9-AS1 was co-transfected into HEK-293T cells with hsa-mir-96 mimic or NC. **(B)** The luciferase reporter plasmid containing wild type (PRDM16-WT) or mutant (PRDM16-MUT) 3’UTR of PRDM16 was co-transfected into HEK-293T cells with hsa-mir-96 mimic or NC (**P < 0.01).

To elucidate the ceRNA network among ADAMTS9-AS1, hsa-mir-96, and its targets in PCa, we conducted luciferase reporter assays. The ADAMTS9-AS1 sequence containing the predicted hsa-mir-96 binding site was cloned downstream of the luciferase gene and named ADAMTS9-AS1 wild-type (WT). The hsa-mir-96 binding site was deleted, resulting in the ADAMTS9-AS1 mutant (MUT). Both WT and MUT were transfected together with hsa-mir-96 mimic. Overexpression of hsa-mir-96 reduced the luciferase activity of the ADAMTS9-AS1 WT reporter vector, but not the ADAMTS9-AS1 MUT vector (**Figure 6A**). In addition, the WT 3’UTR sequence of PRDM16 (containing the predicted hsa-mir-96 binding site) or mutant constructs (containing a deletion in the hsa-mir-96 binding sites) were cloned downstream of the luciferase gene. These plasmids were transfected into HEK293T cells together with control miRNA, hsa-mir-96 mimic. Transfection with hsa-mir-96 mimic reduced the luciferase activity of the PRDM16 reporter vector, but not the mutated vector, indicating that hsa-mir-96 regulates PRDM16 expression in PCa cells by directly binding to the predicted site in the 3’ UTR of PRDM16 (**Figure 6B**). Collectively, these data suggest that ADAMTS9-AS1 modulates the expression of PRDM16 by post-transcriptional regulation of hsa-mir-96.

## Discussion

Recently, many studies have supported a novel regulatory mechanism of lncRNA in human cancers, mainly represented by the interaction between lncRNA and miRNA. LncRNA can act as a ceRNA or molecular sponge to regulate miRNA expression and regulate the occurrence and progression of many cancers (Tu et al., 2019; Ao et al., 2019; Yu et al., 2019). For example, lncRNA Unigene 56159 promotes the epithelial-mesenchymal transition of hepatoma cells by acting as a ceRNA of miR-140-5p (Lv et al., 2016). MEG3 inhibits the invasion of bladder cancer cells by competing with PHLPP2 to bind to miR-27a and negatively regulates c-Myc as a ceRNA (Huang et al., 2019). PVT1 regulates the expression of HK2 in gallbladder cancer cells by competitive binding to miR-143, and controls aerobic glucose metabolism to promote cell proliferation and metastasis (Chen et al., 2019).

Although there have been great advances in therapeutic strategies for PCa, almost all patients develop castration-resistant PCa (Cheng et al., 2018). According to the ceRNA hypothesis, lncRNAs can act as miRNA sponges to construct a complex ceRNA network with miRNAs and mRNAs (Zhou et al., 2014). LncRNA has received extensive attention in recent years, however, miRNAs and mRNAs also deserve more attention. As few studies have reported the role of PRDM16 in PCa, the molecular mechanism of PRDM16 in PCa was the focus of our study. We found that PRDM16 expression was significant for the survival curves of PCa patients with different Gleason scores from TCGA database (Chandrashekar et al., 2017) (P < 0.0001, **Supplementary Figure S2A**). ROC curves for hsa-mir-96 and PRDM16 were generated to distinguish PCa tissue from normal tissue (**Supplementary Figures S2B—C**). hsa-mir-96 and PRDM16 expression exhibited high diagnostic values to distinguish PCa from non-PCa tissues, with an AUC of 0.9338 and 0.7523, respectively. Dysregulated hsa-mir-96 expression is reported to play various roles in tumorigenesis, f.e., colon cancer (Huang and Pan, 2019), lung adenocarcinoma (Zhao et al., 2018), and breast cancer (Lee and Jiang, 2017). Undoubtedly, hsa-mir-96 regulation is crucial in cancer-related signaling pathways.

To explore the molecular mechanism of ceRNA in PCa, we downloaded the gene expression of a large number of PCa patients from the TCGA database, and identified 381 DELs, 35 DEMis, and 689 DEMs using R analysis. Cytoscape was used to perform network visualization for the regulatory relationship of the DELs–DEMis–DEMs network, including 27 DELs, seven DEMis, and three DEMs. Further analysis with GEPIA found that PCAT1, PCA3, and ADAMTS9-AS1 may serve as molecular markers in 27 DELs. Recently, several scholars have indicated that PCAT1 and PCA3 are important genomic biomarkers for PCa. PCAT1 may accelerate PCa cell proliferation, migration, and invasion as well as suppress apoptosis by upregulating FSCN1 *via* miR-145-5p (Xu et al., 2017). PCAT1 has attracted attention as a potential prognostic marker and therapeutic target in multiple types of human cancer (Prensner et al., 2014; Qiao et al., 2017; Cui et al., 2017). The levels of PCA3 and PSA proteolytic activity in prostatic secretions provide an effective pre-surgical biochemical predictor of early PCa recurrence (Jeske et al., 2017). ADAMTS9-AS1 functions as a ceRNA, effectively becoming a sponge for hsa-mir-96 and modulating the expression of PRDM16. Thus, it provides clinicians and patients with independent, clinically useful information to make more informed decisions regarding the need for biopsies. Additionally, we were also surprised to find that ADAMTS9-AS1 acts as a ceRNA that regulates the occurrence and development of tumors in breast cancer (Fan et al., 2018), bladder cancer (Xu et al., 2019), and colon adenocarcinoma (Xing et al., 2018).

To identify novel diagnostic markers for PCa, ADAMTS9-AS1 was selected for further functional assessment with circlncRNAnet. In the present study, GO analysis revealed that a number of GO terms were significant with *P* < 0.05. These significant GO terms involved biological process (BP), cellular component (CC), molecular function (MF), transcription factor (TF), and HM. The pathway analysis revealed the cGMP-PKG signaling pathway, calcium signaling pathway, ras signaling pathway, adrenergic signaling in cardiomyocytes, cAMP signaling pathway, Rap1 signaling pathway, and proteoglycans in cancer. In addition, our data demonstrated that si-ADAMTS9-AS1 promotes cell growth and proliferation. ADAMTS9-AS1 acted as an anti-oncogene ceRNA by binding and sequestering hsa-mir-96 to regulate PRDM16. Moreover, we performed dual luciferase reporter assay to confirm that ADAMTS9-AS1 functions as a ceRNA and competitively binds to hsa-mir-96, thus regulating PRDM16. The main cause of death due to malignant tumors is distant metastases. Competitive endogenous RNAs regulate gene expression and play an important role in the occurrence and development of malignant tumors. LncRNA, as a ceRNA, regulates target genes by competitively binding microRNA, thereby affecting the invasion and metastasis of malignant tumors. Thus, ADAMTS9-AS1 may not only serve as a biomarker, but also as a potential therapeutic target.

In summary, this study revealed the PCa ceRNA expression profile and demonstrated that ADAMTS9-AS1 may serve as a candidate diagnostic biomarker or potential therapeutic target in PCa. Our results may provide a better understanding of the role that the lncRNA–miRNA-mRNA ceRNA network plays during PCa development. This study has provided insight regarding molecular therapeutic strategies for PCa. Nevertheless, our results are preliminary and further studies are needed to explore the biological functions and molecular mechanisms of the ADAMTS9-AS1-hsa-mir-96-PRDM16 ceRNA network in PCa.

## Data Availability Statement

The raw data supporting the conclusions of this article will be made available by the authors, without undue reservation, to any qualified researcher.

## Author Contributions

JW, RC, and SJ wrote the main manuscript text. WM and XjW prepared all figures. XlW and ZH designed the experiments, YJ provided funding for the subject, and all authors reviewed the manuscript.

## Funding

This work was supported by grants from Postgraduate Innovation Research Project of Mudanjiang Medical University (No.2018YJSCX-02MY) and Operation Expenses Research Project for Universities’ basic Scientific Research of Heilongjiang Provincial (No.2018KYYWFMY-0034).

## Conflict of Interest

The authors declare that the research was conducted in the absence of any commercial or financial relationships that could be construed as a potential conflict of interest.
