# Corrigendum: Data Mining and Expression Analysis of Differential lncRNA ADAMTS9-AS1 in Prostate Cancer

**DOI:** 10.3389/fgene.2020.00361

**Published:** 2020-04-17

**Authors:** Jiahui Wan, Shijun Jiang, Ying Jiang, Wei Ma, Xiuli Wang, Zikang He, Xiaojin Wang, Rongjun Cui

**Affiliations:** ^1^Department of Biochemistry and Molecular Biology, Mudanjiang Medical University, Mudanjiang, China; ^2^Department of Clinical Laboratory, Harbin Public Security Hospital, Harbin, China; ^3^Department of Clinical Laboratory, Daqing Medical College, Daqing, China; ^4^Department of Clinical Laboratory, The Seventh Hospital in Qiqihar, Qiqihar, China

**Keywords:** prostate cancer, ADAMTS9-AS1, ceRNA, Hsa-miR-96, PRDM16

In the published article, the order of affiliation 1&2 were incorrect. Also the affiliation “Mudanjiang Medical University” has been added for author Jiahui Wan.

The authors apologize for this error and state that this does not change the scientific conclusions of the article in any way. The original article has been updated.

