# Supplementary material for: Data Mining and Expression Analysis of Differential lncRNA ADAMTS9-AS1 in Prostate Cancer
Source: Front Genet. 2020 Feb 21;10:1377. doi: 10.3389/fgene.2019.01377 (PMC7049946; doi:10.3389/fgene.2019.01377)
Supplement: Supplementary file 1 [file DataSheet_1.docx]

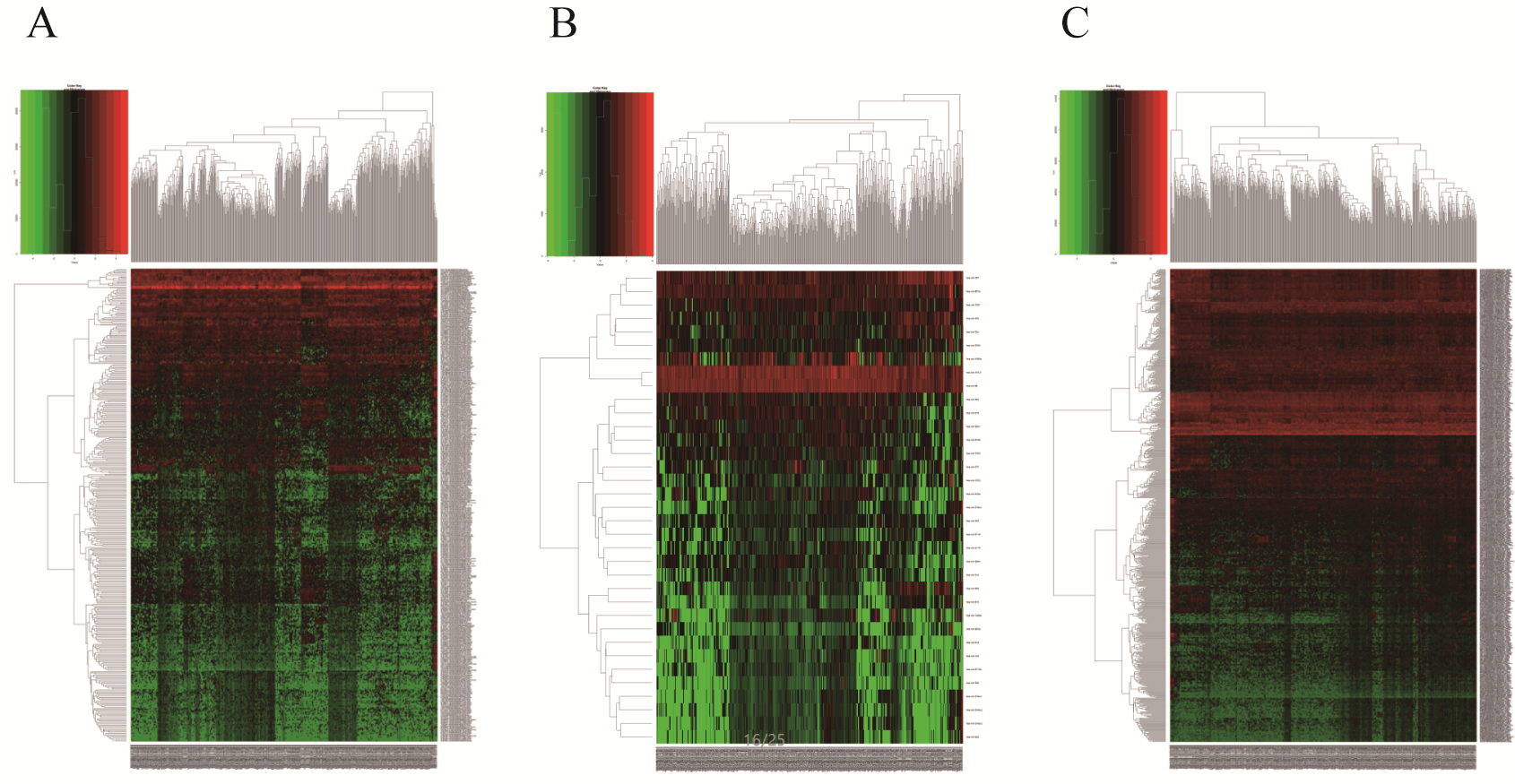


**Supplementary Figure 1.** The heat map of DELs (A), DEMis (B), and DEMs (C) in PCa and adjacent normal prostate tissue.


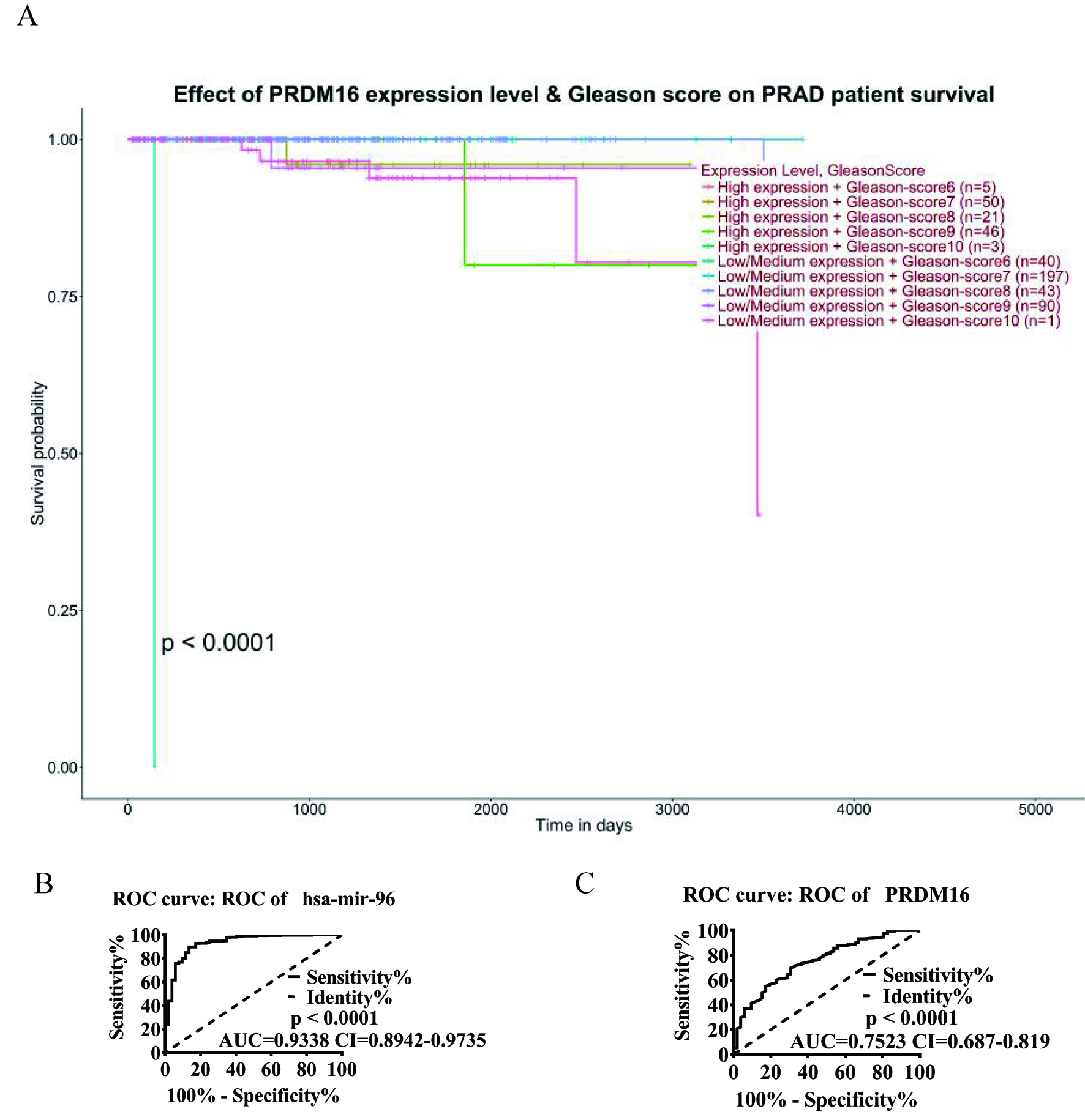


**Supplementary Figure S2:** Relative hsa-mir-96 and PRDM16 expression, ROC curves, and patient survival in prostate cancer. (A) Survival curve of PRDM16 with high and low PRDM16 groups of prostate cancer patients with different Gleason scores from the TCGA database. (B−C) ROC curves of hsa-mir-96 and PRDM16 sorted by AUC in prostate cancer. The solid line represents the sensitivity curve and the dotted line represents the identity line. The x-axis indicates the false positive rate (1-Specificity) and the y-axis indicates the true positive rate (Sensitivity). These curves were provided by GraphPad Prism 5.
